# V367F Mutation in SARS-CoV-2 Spike RBD Emerging during the Early Transmission Phase Enhances Viral Infectivity through Increased Human ACE2 Receptor Binding Affinity

**DOI:** 10.1128/JVI.00617-21

**Published:** 2021-07-26

**Authors:** Junxian Ou, Zhonghua Zhou, Ruixue Dai, Jing Zhang, Shan Zhao, Xiaowei Wu, Wendong Lan, Yi Ren, Lilian Cui, Qiaoshuai Lan, Lu Lu, Donald Seto, James Chodosh, Jianguo Wu, Gong Zhang, Qiwei Zhang

**Affiliations:** a Guangdong Provincial Key Laboratory of Tropical Disease Research, School of Public Health, Southern Medical University, Guangzhou, Guangdong, China; b Key Laboratory of Functional Protein Research of Guangdong Higher Education Institutes, Institute of Life and Health Engineering, College of Life Science and Technology, Jinan University, Guangzhou, Guangdong, China; c Department of Environmental Science and Engineering, Fudan University, Shanghai, China; d Guangdong Provincial Key Laboratory of Virology, Institute of Medical Microbiology, Jinan University, Guangzhou, Guangdong, China; e Novoprotein Scientific Inc., Shanghai, China; f Key Laboratory of Medical Molecular Virology (MOE/NHC/CAMS), School of Basic Medical Sciences, Shanghai Public Health Clinical Center, Fudan University, Shanghai, China; g Bioinformatics and Computational Biology Program, School of Systems Biology, George Mason University, Manassas, Virginia, USA; h Department of Ophthalmology, Howe Laboratory, Massachusetts Eye and Ear, Harvard Medical School, Boston, Massachusetts, USA; Loyola University Chicago

**Keywords:** COVID-19, SARS-CoV-2, receptor-binding domain (RBD), viral infectivity, ACE2 receptor, variants, mutation

## Abstract

The current pandemic of COVID-19 is caused by a novel coronavirus, severe acute respiratory syndrome coronavirus 2 (SARS-CoV-2). The SARS-CoV-2 spike protein receptor-binding domain (RBD) is the critical determinant of viral tropism and infectivity. To investigate whether naturally occurring RBD mutations during the early transmission phase have altered the receptor binding affinity and infectivity, we first analyzed *in silico* the binding dynamics between SARS-CoV-2 RBD mutants and the human angiotensin-converting enzyme 2 (ACE2) receptor. Among 32,123 genomes of SARS-CoV-2 isolates (December 2019 through March 2020), 302 nonsynonymous RBD mutants were identified and clustered into 96 mutant types. The six dominant mutations were analyzed applying molecular dynamics simulations (MDS). The mutant type V367F continuously circulating worldwide displayed higher binding affinity to human ACE2 due to the enhanced structural stabilization of the RBD beta-sheet scaffold. The MDS also indicated that it would be difficult for bat SARS-like CoV to infect humans. However, the pangolin CoV is potentially infectious to humans. The increased infectivity of V367 mutants was further validated by performing receptor-ligand binding enzyme-linked immunosorbent assay (ELISA), surface plasmon resonance, and pseudotyped virus assays. Phylogenetic analysis of the genomes of V367F mutants showed that during the early transmission phase, most V367F mutants clustered more closely with the SARS-CoV-2 prototype strain than the dual-mutation variants (V367F+D614G), which may derivate from recombination. The analysis of critical RBD mutations provides further insights into the evolutionary trajectory of early SARS-CoV-2 variants of zoonotic origin under negative selection pressure and supports the continuing surveillance of spike mutations to aid in the development of new COVID-19 drugs and vaccines.

**IMPORTANCE** A novel coronavirus, severe acute respiratory syndrome coronavirus 2 (SARS-CoV-2), has caused the pandemic of COVID-19. The origin of SARS-CoV-2 was associated with zoonotic infections. The spike protein receptor-binding domain (RBD) is identified as the critical determinant of viral tropism and infectivity. Thus, whether mutations in the RBD of the circulating SARS-CoV-2 isolates have altered the receptor binding affinity and made them more infectious has been the research hot spot. Given that SARS-CoV-2 is a novel coronavirus, the significance of our research is in identifying and validating the RBD mutant types emerging during the early transmission phase and increasing human angiotensin-converting enzyme 2 (ACE2) receptor binding affinity and infectivity. Our study provides insights into the evolutionary trajectory of early SARS-CoV-2 variants of zoonotic origin. The continuing surveillance of RBD mutations with increased human ACE2 affinity in human or other animals is critical to the development of new COVID-19 drugs and vaccines against these variants during the sustained COVID-19 pandemic.

## INTRODUCTION

A novel coronavirus, severe acute respiratory syndrome coronavirus 2 (SARS-CoV-2), has caused outbreaks of coronavirus disease 2019 (COVID-19) globally beginning in mid-December 2019, with an epicenter in Wuhan, China (1–3). As of 9 April 2021, SARS-CoV-2 had infected 133 million people worldwide and caused 2.8 million deaths, with an estimated fatality rate of 2.2% (4). This on-going pandemic of COVID-19 has become the most serious threat to public health in this century.

The origin of SARS-CoV-2 remains elusive. However, the initial cases were largely associated with a seafood market, which indicated potential zoonotic transmissions (1, 5, 6). Although bats and pangolins are most likely the reservoir and the intermediate hosts in the wild, more evidence is needed to support zoonotic transmission and to track the origin of this new coronavirus (5, 6).

Angiotensin-converting enzyme 2 (ACE2) is the host cellular receptor for the SARS-CoV-2 spike glycoprotein, which is similar to its counterpart in SARS-CoV. The receptor-binding domain (RBD) of the spike protein subunit S1 interacts directly with ACE2, providing for tight binding to the peptidase domain of ACE2 (7–10). Therefore, RBD is the critical determinant of virus-receptor interaction and reflects viral host range, tropism, and infectivity. Although the RBD sequences of different SARS-CoV-2 strains circulating globally are largely conserved, mutations have appeared; these may account for differences in viral infectivity and also contribute to its spread (10–14).

Meanwhile, S glycoprotein participates in antigenic recognition and is expressed on the virion surface, likely to be immunogenic, carrying both T-cell and B-cell epitopes. The potential antibody binding sites that have been identified indicate RBD has important B-cell epitopes. The main antibody binding sites substantially overlap the RBD, and the antibody binding to these sites is able to block viral entry into cells (14–16).

To investigate whether mutations in RBD that emerged during the early transmission phase have altered the receptor binding affinities and whether these isolates may have been selected for higher infectivity, the binding dynamics and the infectivity between the SARS-CoV-2 RBD mutants and human ACE2 receptor were modeled and assessed computationally. The evolutionary trajectory of SARS-CoV-2 during the early transmission phase under negative selection pressure was also analyzed. In addition, experimental validation of the enhanced affinity and infectivity of the V367F mutant was performed.

(This article was submitted to an online preprint archive [17].)

## RESULTS

### SARS-CoV-2 spike and RBD mutation mapping and scanning during the early transmission phase.

**FIG 1:**
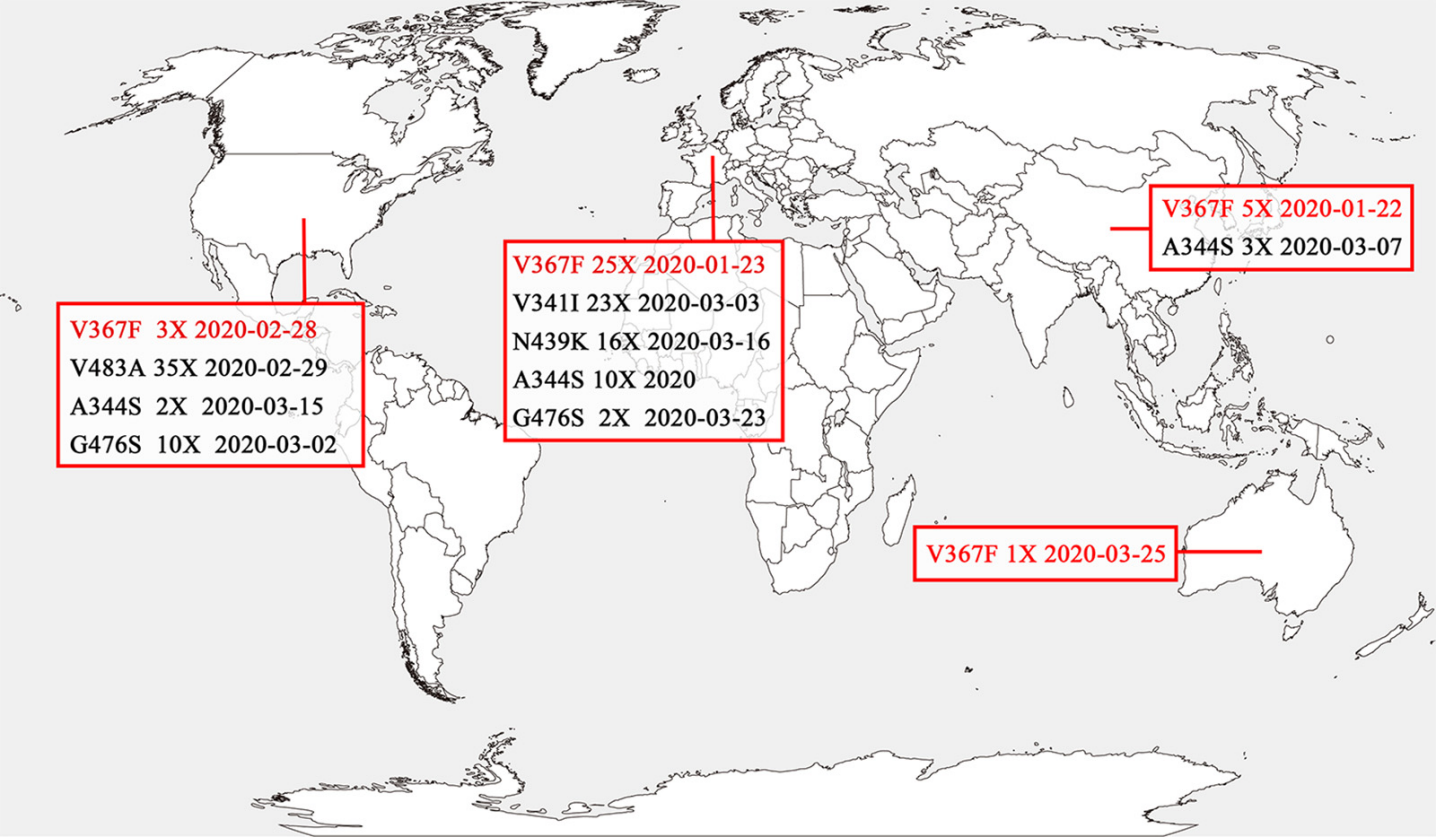
Geographical distribution of the SARS-CoV-2 RBD mutants. The geographic distribution of the RBD mutants on four continents (>10 isolates) is displayed. The mutants marked in black are mutants with similar binding affinities as strain Wuhan-Hu-1. V367F mutants with the enhanced binding affinity were found on all of the four continents and are marked in red. The mutants analyzed were isolated as of 31 March 2020.

In total, 32,123 SARS-CoV-2 isolates with whole-genome sequences available in public databases and sampled before 31 March 2020 were analyzed. Among them, 302 isolates with RBD mutations were identified compared with reference strain Wuhan-Hu-1 that was the first isolated in Wuhan in December 2019. All the mutants were clustered into 96 mutant types, six of which were dominant mutant types that were found in more than ten isolates (Table 1): V483A (35×), V367F (34×), V341I (23×), N439K (16×), A344S (15×), and G476S (12×). V483A and V367F accounted for 11.59% and 11.26% of 302 mutants, respectively. V367F mutants occurred on 22 January 2020, which was the earliest dominant mutant type. The detailed alignments of amino acid sequences in the RBD of the mutants are shown in Fig. S1 in the supplemental material. These mutants were emergent in multiple continents, including Asia, Europe, North America, and Oceania. Most RBD mutants were circulating in Europe and North America (Fig. 1). V367F mutants were found on all four continents.

**TABLE 1:** Dominant mutations in the spike RBD (>10 isolates) emerging during the early transmission phase (December 2019 through March 2020) and their binding affinity change^*a*^

Amino acid position in spike gene	Amino acid change	% of mutants	Date of occurrence (yr/mo/day)	ID of the first mutant	No. of dominant mutations	Binding free energy increase
483	V to A	11.59	2020/2/29	EPI_ISL_417159	35	No
367	V to F	11.26	2020/1/22	EPI_ISL_408975	34	Yes
341	V to I	7.95	2020/3/3	EPI_ISL_454450	23	No
439	N to K	5.30	2020/3/16	EPI_ISL_425684	16	No
344	A to S	4.97	2020/3/7	EPI_ISL_506954	15	No
476	G to S	3.97	2020/3/2	EPI_ISL_417081	12	No

a The numbers of dominant mutations in the spike RBD (as of 31 March 2020) are shown. The date of mutant occurrence and ID of the first mutant are identified. Receptor binding efficiencies of dominant mutants were evaluated by both amino acid property and binding free energy change using the MM-PBSA method (35, 36). The numbers of total mutants emerging during the early transmission phase are 302. Among them, dominant mutations that emerged in >10 isolates are shown.

### Nucleotide diversity and selective pressure on SARS-CoV-2 spike gene and RBD.

**FIG 2:**
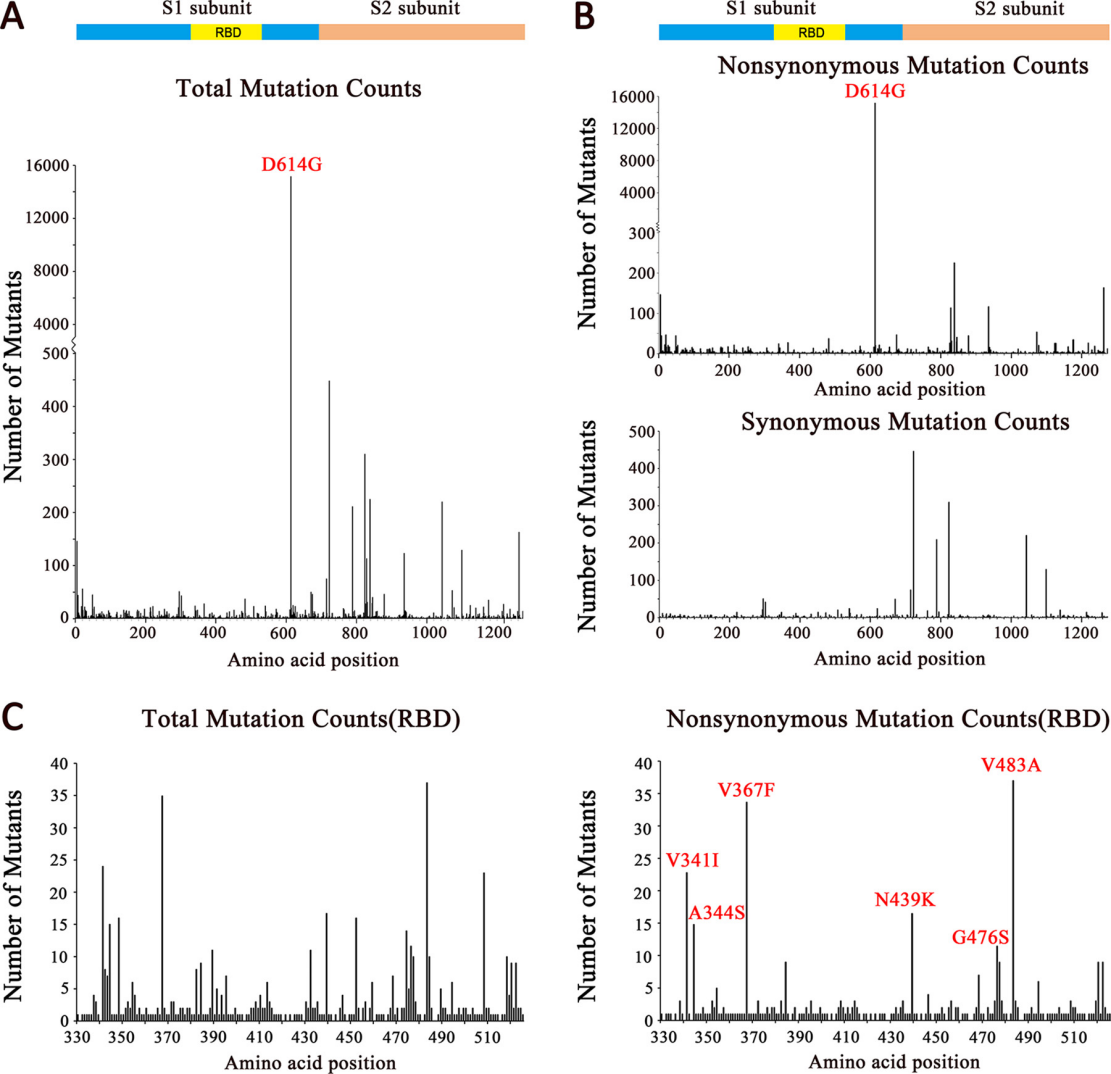
Mutation scanning graphs of the SARS-CoV-2 S gene. Mutation sites compared to strain Wuhan-Hu-1 were analyzed by BioAider (V1.314) (29). (A) Total mutations (synonymous and nonsynonymous) in the spike gene. (B) Nonsynonymous and synonymous mutations in the spike gene. (C) Total mutations and nonsynonymous mutations in the RBD. All positions containing gaps and missing data were eliminated. Structural domains are annotated. The peak signals of D614G and the other dominant mutations are marked in red. The ordinates show the numbers of mutants. The isolates analyzed were isolated as of 31 March 2020.

As SARS-CoV-2 S protein mediates attachment of the virus to cell surface receptors and fusion between virus and cell membranes, the polymorphism and divergence of the S gene were analyzed by DnaSP6 (version 6.12.03) (37). Overall, the S2 subunit was more diverse than the S1 subunit (Fig. 2A). The D614G mutation in the S1 subunit accounted for most of the polymorphism and divergence. Most of the other synonymous and nonsynonymous mutations occurred in the S2 subunit (Fig. 2B), of which the number of synonymous mutations was more than that of nonsynonymous mutations. During the early transmission phase, RBD was conserved and not as diverse as the S2 subunit (Fig. 2A and B). The six major mutant types are shown in Fig. 2C.

Since RBD is the only domain to bind the human ACE2 receptor and, in turn, initiates cell entry, it is believed that the RBD should be highly conserved. To test this hypothesis, we investigated the selective pressures on the S gene during the early transmission phrase by calculating the nonsynonymous/synonymous substitution rate ratios (*dN/dS* ratios) for various segments of the S gene from the 32,123 SARS-CoV-2 isolates (18, 19). The entire S gene exhibited a *dN/dS* of 0.86, close to the 0.83 reported previously (20) (Table 2). However, different regions of the S gene may be subject to different selection pressures. The S1 gene exhibited a high *dN/dS* (2.05) due to the widely spreading nonsynonymous mutation D614G in the S1 gene (21). The S2 gene exhibited a lower *dN/dS* (0.2388551), indicating that the S2 gene was more conserved than S1 under negative selection. The S1-RBD showed a lower *dN/dS* (0.7258567) than the entire S gene (0.8569338). Therefore, the functional relevance of the RBD mutations may be inferred.

**TABLE 2:** Nucleotide substitution rates and selection pressures for S gene^*a*^

Gene	Length (bp)	Mean no. of substitutions per site		*dN/dS*
		Nonsynonymous	Synonymous	
S	3822	0.0002342	0.0002733	0.8569338
S1	2043	0.0003519	0.0001715	2.0518950
S1-RBD	585	0.0000699	0.0000963	0.7258567
S2	1779	0.0000943	0.0003948	0.2388551

a The numbers of nonsynonymous and synonymous differences per sequence from averaging over all sequence pairs are shown. Analyses were conducted using the Nei-Gojobori method (Jukes-Cantor model) in Mega X (10.0.2) (18, 19). The analyses involved 32,123 SARS-CoV-2 S gene sequences. All positions containing gaps and missing data were discarded.

### V367F mutant emergent during the early transmission phase binds human ACE2 receptor with higher affinity.

**FIG 3:**
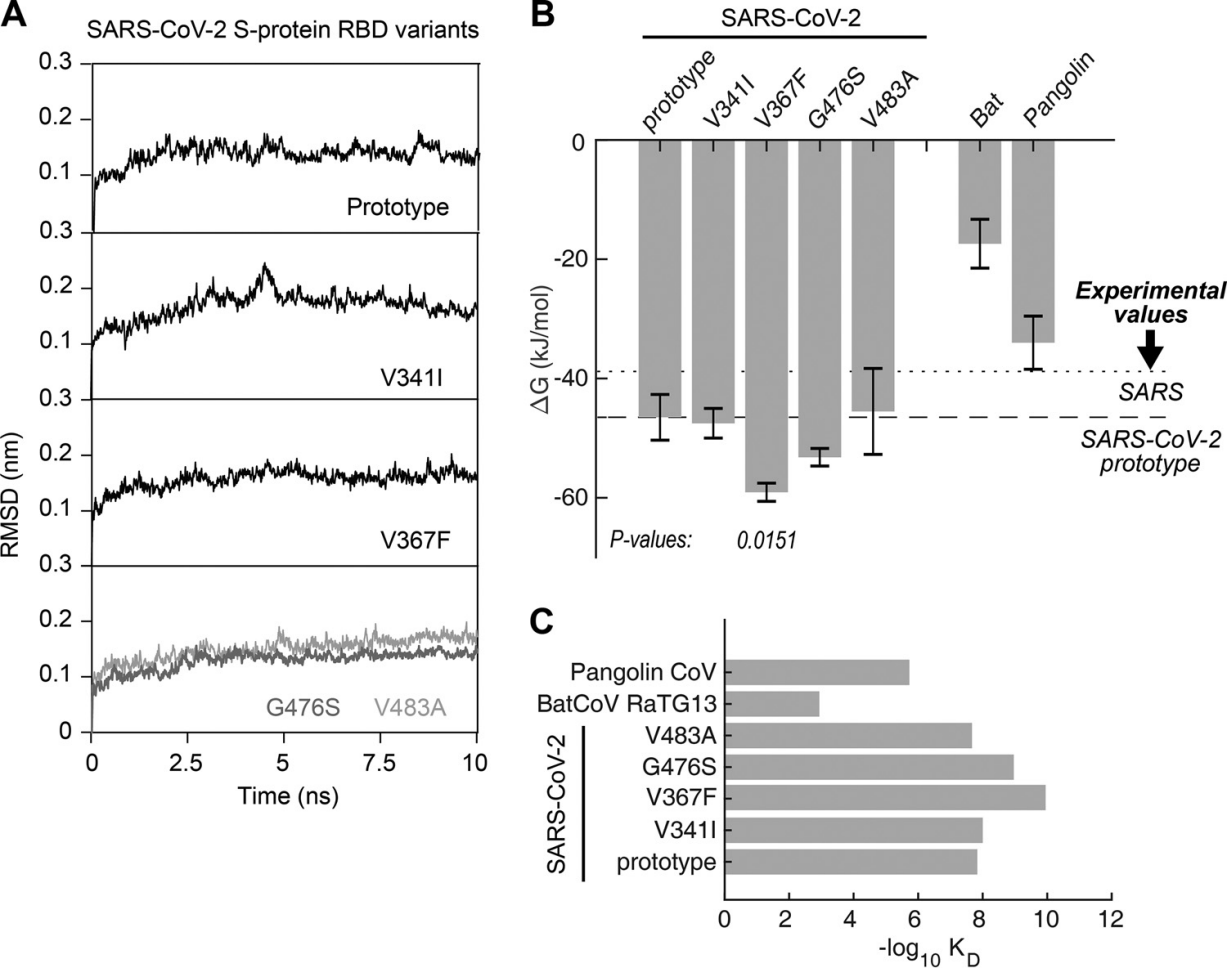
Binding free energy calculated for the SARS-CoV-2 S-RBD to human ACE2 receptor. (A) RMSD of typical MD trajectories of the SARS-CoV-2 prototype and the mutants. (B) Comparison of the binding free energy (Δ*G*) of the RBDs of human SARS-CoV-2, bat CoV, and pangolin CoV to the human ACE2. Note the Δ*G* is inversely proportional to the binding affinity. Data are presented as means ± standard deviations (SDs). *P* values were calculated using single-tailed Student's *t* test. The *P* values are shown for those with a *P* value of <0.05. The Δ*G* calculated from experimental *K_D_* values of SARS and the SARS-CoV-2 prototype are marked in dotted and dashed lines, respectively. (C) Comparison of the equilibrium dissociation constants (*K_D_*) calculated with the Δ*G*.

To estimate the functional changes suggested by the RBD mutations, we performed molecular dynamics (MD) simulations for the prototype SARS-CoV-2 (Wuhan-Hu-1 strain) and the RBD mutants in order to assess their binding energy to the human ACE2 receptor. Each simulation was performed at 10 ns, and each model was simulated in triplicates. All trajectories reached a plateau of root mean square deviation (RMSD) after 2 to 5 ns (Fig. 3A), indicating that their structures reached equilibrium. All of the subsequent computations on thermodynamics were based on the 5- to 10-ns trajectories. The Δ*G* of the V367F mutant was significantly low (∼60 kJ/mol) (*P* = 0.0151), approximately 25% lower than for the prototype strain (−46.5 kJ/mol, calculated from the experimentally measured equilibrium dissociation constant [*K_D_*]) (Fig. 3B), suggesting a significantly increased affinity to human ACE2; the other mutants showed a similar Δ*G* to that of the prototype (Fig. 3B). Compared to the *K_D_* (14.7 nM) of the prototype RBD, the *K_D_* of the V367F mutant was calculated as 0.11 nM (Fig. 3C), which was 2 orders of magnitude lower than for the prototype strain, indicating an increased affinity to the human ACE2 receptor. In comparison, the bat CoV RBD (strain RaTG13, with the highest genome similarity) showed a much lower binding affinity (*K_D_* = 1.17 mM; Δ*G* = −17.4 kJ/mol) to human ACE2 than the pangolin CoV (*K_D_* = 1.89 μM; Δ*G* = −33.9 kJ/mol). The affinity of the pangolin CoV to human ACE2 was lower than that of the SARS-CoV-2 prototype strain (*K_D_* = 14.7 nM; Δ*G* = −46.5 kJ/mol) (Fig. 3B and C).

### Structural basis for the increased affinity of the SARS-CoV RBD mutants.

**FIG 4:**
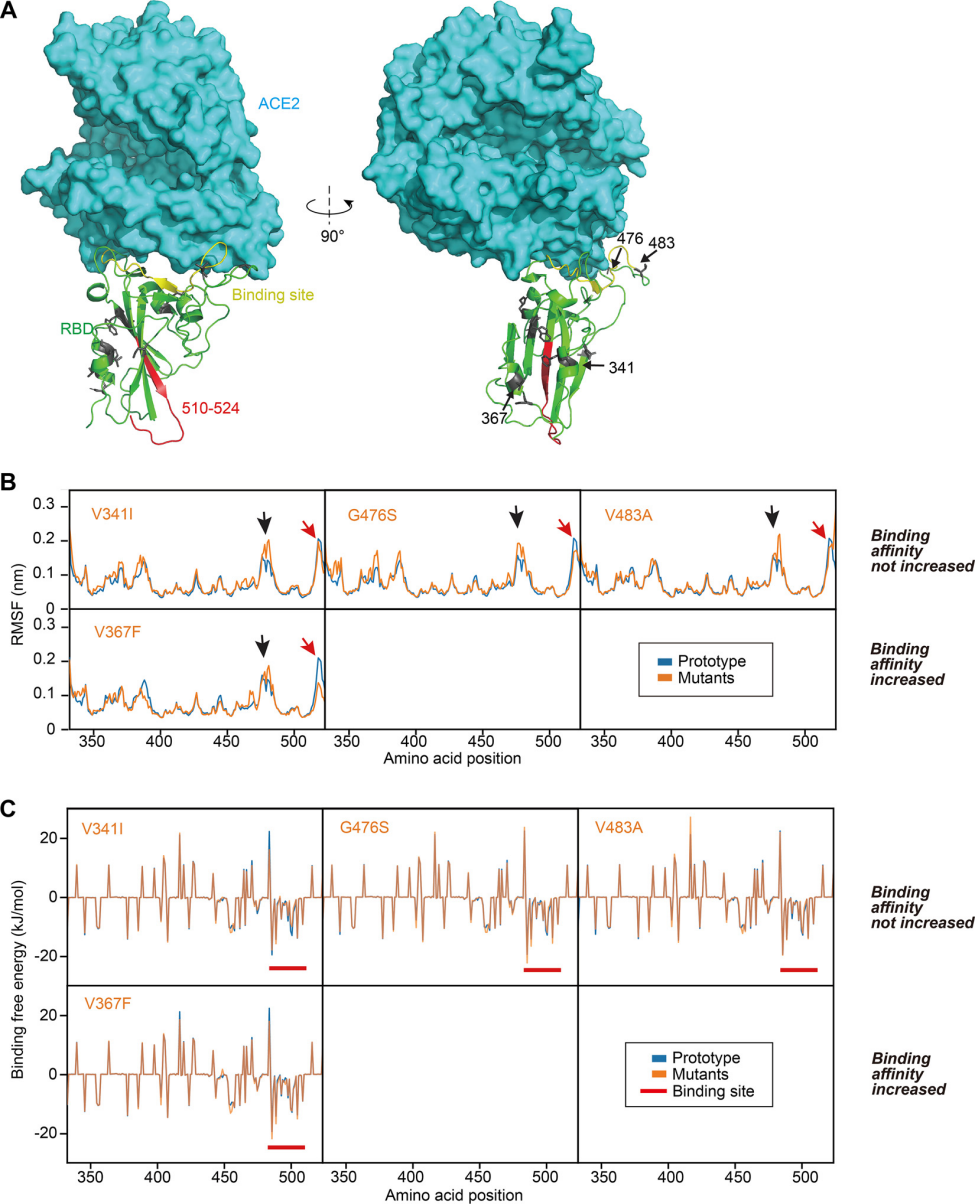
Structural analysis of RBD mutants and the effects on their binding affinity. (A) Binding surface and interaction of the RBD to ACE2, with the locations of the mutant amino acids noted. Beta-sheet structure scaffold was centered by residues 510 to 524 (in red). (B) Root mean square of fluctuation (RMSF) values of the mutants were compared to that of the prototype. Red arrows denote the fragment of residues 510 to 524. Black arrows denote the fragment of residues 475 to 485. (C) Contribution of each amino acid to the binding free energy. Red bars denote the binding site.

To elucidate the structural basis of the increased affinity of the V367F mutant, we investigated the dynamics of the residues in these structures in greater detail. The binding surface of the RBD to ACE2 is largely arrayed in a random coil conformation, which lacks structural rigidity. Logically, a firm scaffold should be necessary to maintain this conformation of the interaction surface and thus may facilitate the binding affinity. The beta-sheet structure scaffold, centered by residues 510 to 524 (Fig. 4A, in red), apparently provides this rigidity. “Higher-affinity” mutant V367F showed a considerable decrease of the root mean square of fluctuation (RMSF) at this region, demonstrating a more rigid structure; this was not observed for other mutants (Fig. 4B). Coincidentally, the substitutions that account for the affinity increase of V367F are all located near this fragment. Indeed, residues 475 to 485, which is a random coil near the binding site, showed a remarkably higher RMSF for the “similar-affinity” mutants than the higher-affinity V367F mutant (Fig. 4B). Moreover, the higher-affinity mutant V367F exhibited a generally decreased Δ*G* in the binding site region in contrast to that of the similar-affinity mutants (Fig. 4C).

### Experimental validation of the enhanced affinity and infectivity of the V367F mutant.

**FIG 5:**
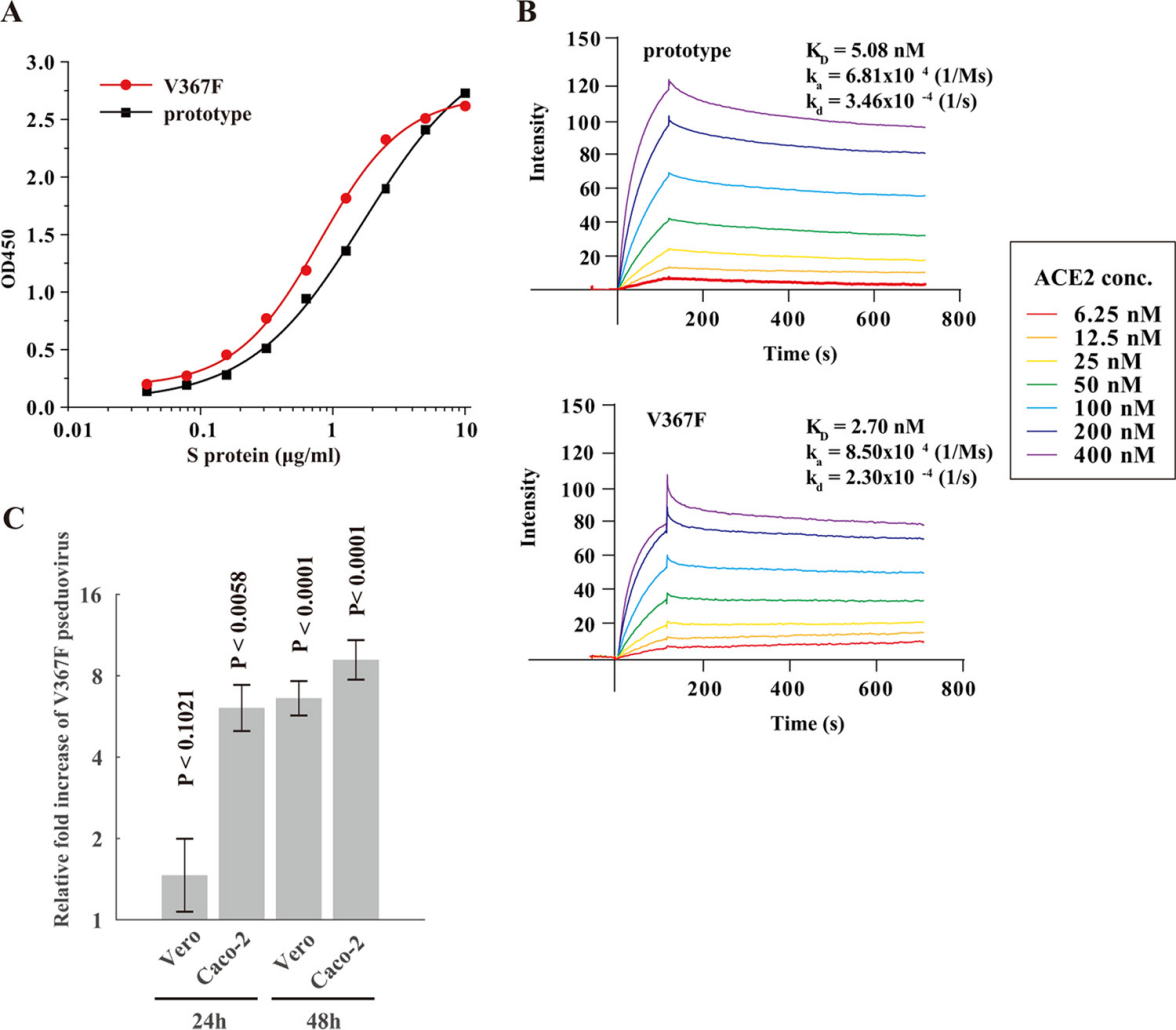
Experimental validation of the enhanced affinity and infectivity of the V367F mutant. (A) Comparison of the binding affinity of prototype S protein and V367F mutant to human ACE2 receptor by ligand-receptor binding ELISA. (B) Comparison of the binding affinity of prototype S protein and V367F mutant to human ACE2 protein by SPR. (C) Quantification of the genome copy number of the V367F mutant versus the prototype using pseudovirus infection assay. The relative fold increases of viruses infecting the cells are shown by the pseudoviral DNA copy number of the V367F mutant in both Vero and Caco-2 cells at 24 h p.i. and 48 h p.i. Experiments were performed in triplicates, and the *P* values were calculated using two-tailed *t* test for two samples with different variances.

As of 31 March 2020, among all of the mutants with dominant mutations in spike RBD (>10 isolates), most mutations resulted in a substitution of amino acids with similar properties. V367F is the only mutant with a higher binding affinity, as calculated by MD simulation (Fig. 3). Therefore, the binding affinity and the infectivity of the V367F mutant were further validated experimentally. First, we performed experiments to assess the binding affinity *in vitro* with a receptor-ligand binding enzyme-linked immunosorbent assay (ELISA) using purified S proteins and human ACE2 protein. The result showed that the V367F mutation lowered the 50% effective dose (ED_50_) concentration (ED_50_ = 0.8 ± 0.04 μg/ml) compared to that of the prototype (ED_50_ = 1.7 ± 0.14 μg/ml) (Fig. 5A). This demonstrates that the V367F mutant has a higher affinity to human ACE2 than the prototype. Second, we performed surface plasmon resonance (SPR) experiments, which yielded the same conclusion: the prototype had a *K_D_* of 5.08 nM compared to the V367F mutant with a *K_D_* of 2.70 nM (Fig. 5B). Additionally, we performed a virus-cell interaction experiment to investigate the invasion efficiency of S proteins using an HIV backbone pseudovirus assay. ACE2-overexpressing Vero and Caco-2 cells were infected by the pseudoviruses bearing the mutant or wild-type RBD with the same multiplicity of infection (MOI). A higher infection efficiency is represented by the increased copy number of the integrated lentivirus genome. At 24 h postinfection (h p.i.), the V367F pseudoviruses showed 6.08× higher copy numbers than the prototype in Caco-2 cells (*P* < 0.01). At 48 h p.i., the V367F pseudoviruses showed 6.61× and 9.16× higher copy numbers than the prototype in Vero (*P* < 0.0001) and Caco-2 cells (*P* < 0.0001), respectively (Fig. 5C). Therefore, the computation and protein and cell validations were consistent with each other: the V367F mutant had enhanced affinity and infectivity.

### Convergence of SARS-CoV-2 RBD and D614G mutations in dominant mutation isolates.

The D614G mutation is located in the S1 region and is outside the RBD of the SARS-CoV-2 spike protein. It has been confirmed that the D614G mutants, which have spread widely, increase virus infectivity by elevating its sensitivity to protease (21). The V367F mutants were initially discovered in January 2020 in Hong Kong. Afterwards, the V367F mutants emerged mainly in Europe, including the United Kingdom, the Netherlands, Austria, and Iceland as well as in the United States, Australia, and China. The D614G+V367F dual mutant initially emerged in March 2020 in the Netherlands (see Table S1).

**FIG 6:**
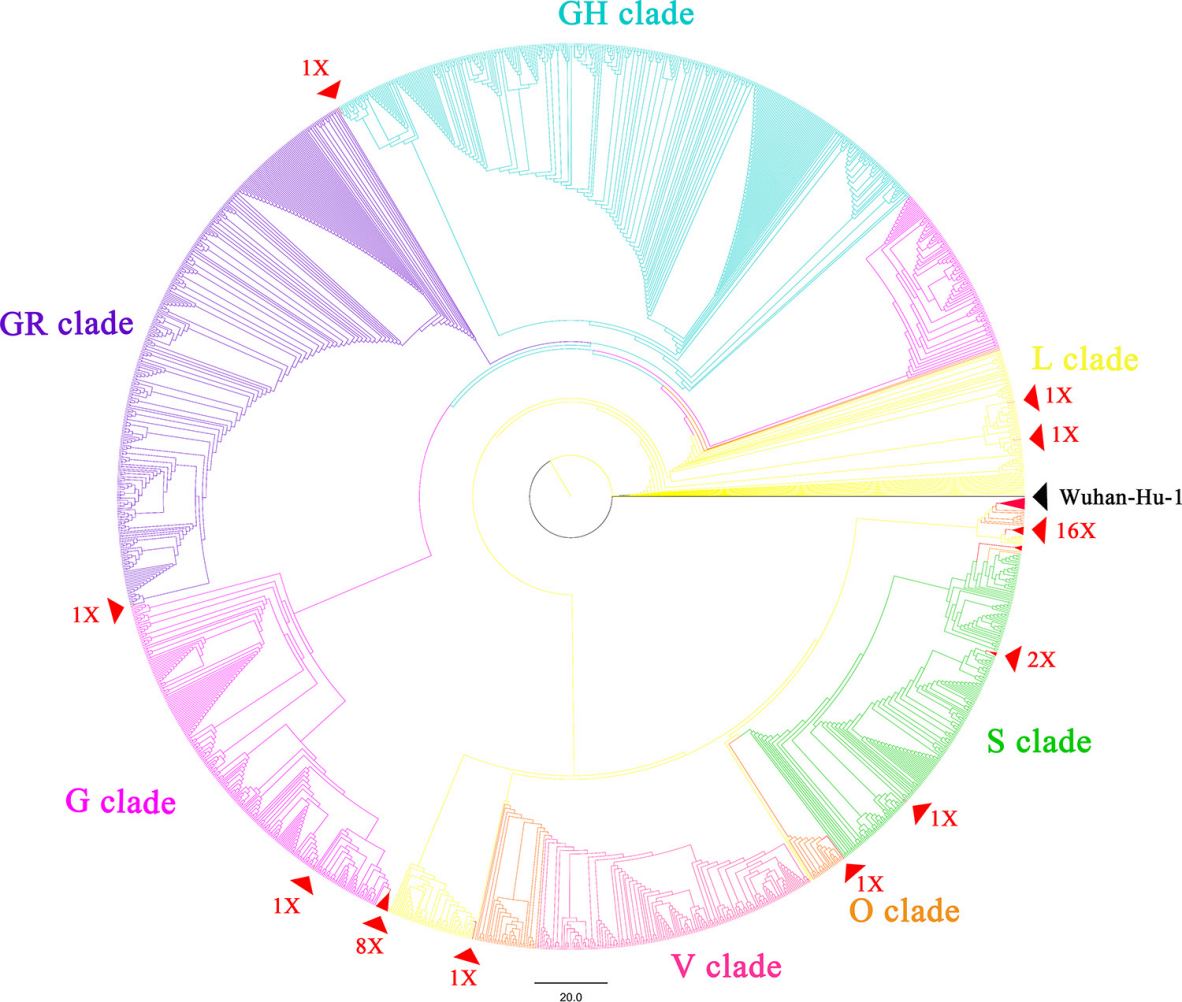
Whole-genome phylogenetic analysis of the SARS-CoV-2 variants emerging during the early transmission phase (December 2019 through March 2020). The whole-genome phylogenetic tree was constructed by IQ-Tree 2.02 using the maximum likelihood method with GTR+F+R3 model, 1,000 bootstrap replicates, and applying default parameters. All 34 V367F mutants were included. For reference, the branch of Wuhan-Hu-01 is marked in black. V367F mutants are marked with red triangles; sampled referenced sequences are annotated in different colors by clades using Figtree 1.4.4 (https://github.com/rambaut/figtree/releases).

The phylogenetic analysis of the V367F mutant genomes during the early transmission phase showed that V367F mutants clustered more closely with the SARS-CoV-2 prototype strain in the L clade. Intriguingly, all of the dual-mutation variants (V367F+D614G) emerging later formed a distinct subcluster in GH, GR, and G clades, separate from the L and S clades (22) (Fig. 6). Compared with all the single-mutation (V367F) variants, dual-mutation (V367F+D614G) variants were emergent in both GH and GR clades. Multiple dual-mutation (V367F+D614G) variants located in different clades had mutations undetected in early V367F mutants. This indicates that the dual mutations may have evolved through several individual evolution events (Fig. 6).

## DISCUSSION

Due to the challenging and on-going pandemic and given the evolving nature of the SARS-CoV-2 virus globally, identifying changes in viral infectivity has proved crucial to restraining the COVID-19 pandemic. Quarantine policies need to be adapted with respect to the viral infectivity change. It is always a dilemma of quarantine and economy. Any government would balance the lost due to the quarantine lockdown versus the lost due to the disease. Numerous models have been raised to estimate the costs. For example, if the variants have evolved to be more infectious, more stringent lockdown measures would be expected.

In our study, we analyzed 32,123 genomes of SARS-CoV-2 isolates (December 2019 through March 2020) and identified 302 nonsynonymous RBD mutants, which clustered into 96 mutant types. The six dominant mutations were analyzed by applying molecular dynamics simulations. The mutant type V367F continuously circulating worldwide displayed higher binding affinity to human ACE2 due to the enhanced structural stabilization of the RBD beta-sheet scaffold. The molecular dynamics simulations also indicated that it would be difficult for bat SARS-like CoV to infect humans. However, the pangolin CoV is potentially infectious to humans. The increased infectivity of V367 mutants was further validated by receptor-ligand binding ELISA, surface plasmon resonance, and pseudotyped virus assays. Phylogenetic analysis of the genomes of V367F mutants showed that during the early transmission phase, most V367F mutants clustered more closely with the SARS-CoV-2 prototype strain than the dual-mutation variants (V367F+D614G) which emerged later and formed a distinct subcluster.

Compared to that of the prototype strain Wuhan-Hu-1, the Δ*G* of the V367F mutation decreased ∼25%. The variant binds to ACE2 more stably due to the enhancement of the base rigidity of the S protein structure. Along with the ongoing adaptation to transmission and replication in humans, some critical mutations in the RBD may boost the binding affinity and lead to an increase in the basic reproduction number (R0).

A previous study found some RBD mutations that enhanced ACE2 affinity but were not selected in pandemic isolates (23). For example, through deep mutation scanning, an assumed N501F mutation was confirmed to have enhanced binding efficiency while not emergent, but later, the N501Y mutation in the same position with a similar enhancing effect was frequently detected in emerging lineage B1.1.7 variants (24). This may partly explain why V367F has not been dominant in the subsequent epidemic. The V367F mutation was also confirmed with higher protein expression level and enhanced stability due to the higher melting temperature in mammalian-expressed purified RBD than the prototype RBD (23).

The V367F mutants were frequently found during the early transmission phase (Table 1). As RBD is conserved in SARS-CoV-2, the coincidence of V367F mutants across large geographic distances indicates that this mutation is more robust and that these variants originated as a novel sublineage, given the close isolation dates (22 and 23 January 2020, respectively). The asymptomatic individuals with the same mutation were “superinfecting” travelers. Along with the epidemiological data, mutation surveillance is of critical importance, as it can reveal more exact transmission routes of the epidemic and provide early warnings of additional outbreaks. Emergence of SARS-CoV-2 RBD mutants with higher binding affinity to the human ACE2 receptor in Hong Kong, France, and other countries during the early transmission phase suggests an increased risk of emergence of more and more variants with “high affinity” and increased infectivity during a sustained pandemic of COVID-19, particularly if effective precautions are not completely implemented.

By tracking the V367F mutation type in the SARS-CoV-2 circulating strains, most of these mutants were first detected within the prototype D614 strains, then together with the G614 variants. This is possibly due to multiple individual recombination events between the D614 strains and G614 mutants, although it is difficult to determine the exact recombinant positions or time point due to the high sequence identities among SARS-CoV-2 variants. D614G is distinct from the RBD mutations: it is not located in the RBD but enhances viral infectivity by elevating its sensitivity to proteases and increasing stability (12, 13, 21). Furthermore, D614G and V367F may function independently and have synergistic effects on viral infectivity. Recombination is known to play an important role in natural coronavirus evolution, which may contribute to the convergence of dual enhancing mutants (D614G+V367F). The phylogenetic and substitution analyses indicated that the divergent clade of dual-mutation mutants was possibly from multiple individual recombination events which introduced multiple mutations. Whether these introduced multiple mutations may produce more adaptive variants needs further study. More attention should be paid to the risk of the accumulation of evolutionary advantages through recombination among the variants.

By tracking dominant RBD mutants up to 31 March 2020, multiple mutations from more than 10 isolates putatively related to host receptor binding and affinity were detected in this study. The equivalent amino acid positions in more than 500 SARS-CoV and Middle East respiratory syndrome coronavirus (MERS-CoV) genomes were compared. Among them, V483A in MERS-CoV and N439K in SARS-CoV resulted in reduced host receptor binding (23, 25). Given the alanine shares similar chemical and structural properties with serine, the A344S variant was expected to have similar affinity to human ACE2 as the prototype strain, which was later confirmed through scanning (23). By tracking these recent variants, we find that only the V367F mutation type was detected continually; the other enhanced mutations have disappeared. This may be because they failed to compete with the dominant D614G variants (12, 21). Interestingly, we discovered some V367F variants combined with the D614G mutation. The recent emerging variants B.1.1.7, B.1.351, and B.1.429, which are currently threatening the world, also partially harbor the V367F mutation, possibly deriving from other recombination events. Whether these multiple recombinations cause the variants to be more infectious, more stable, or better at immune escape requires more study.

The origin of this virus has been of considerable interest and speculation since the outbreak. Due to the high sequence similarity of the bat SARS-like CoV genome and the pangolin CoV RBD sequence to the SARS-CoV-2 sequences, these hosts were thought to have initiated the infection in humans (5, 6). Our results provide more clues to support this hypothesis: the binding energy of the bat SARS-like CoV RBD is too high to bind human ACE2 effectively (*K_D_* in millimolar range). In contrast, the pangolin CoV showed a binding *K_D_* for human ACE2 at the micromolar range, much higher than that of SARS-CoV-2 and just ∼6× higher than that of human SARS-CoV (*K_D_* = 0.326 μM), which indicates that the pangolin CoV has the potential to infect humans in unprotected close contact. Alignments of the genomic sequences of SARS-CoV-2 and pangolin CoVs suggest recombination events, particularly in the RBD domain, between pangolin and bat viruses.

In summary, we have identified 302 RBD mutants clustering into 96 dominant mutant types during the early transmission phase. Among the six dominant mutation types, V367F mutants that emerged in Asia and Europe displayed enhanced structural stability of the spike protein along with higher binding affinities to the human ACE2 receptor. V367F mutants were circulating along with the D614G mutants. This suggests that the V367F mutants are stable and have acquired increased infectivity for humans during the COVID-19 pandemic. The emergence of dual mutants (V367F+D614G) and other combined mutations possibly due to recombination may hint toward the emergence of other variants with increased infectivity or with enhanced escape from the host immune response. Our findings support the continuing surveillance of spike mutations to aid in the development of new COVID-19 drugs and vaccines.

## MATERIALS AND METHODS

### Genome sequence data set in this study.

Full-length gene sequences of SARS-CoV-2 were downloaded from the NCBI GenBank database, and the GISAID EpiFlu database (http://www.GISAID.org). In total, 34,702 SARS-CoV-2 full-genome sequences isolated during early transmission phase (samples collected before 31 March 2020) were downloaded, and the sequences with amino acid mutations in the S protein and RBD region were parsed. The genome sequences with either the V367F mutation or the V367F/D614G dual mutations in the S protein RBD were screened and analyzed in this study (see Table S1 in the supplemental material). For evolution analysis, 1,753 full-genome sequences from each NextStrain (26) clade were filtered and cluster random sampled from the GISAID EpiFlu database.

### Mutation analyses and phylogenetic analyses.

Alignment of S protein sequences from different sources and comparison of ACE2 proteins among different species were performed using MAFFT version 7 with default parameters (https://mafft.cbrc.jp/alignment/server/) and BioEdit (27, 28). Selection pressure analyses were conducted using the Nei-Gojobori method (Jukes-Cantor model) with Mega X (version 10.0.2) (18, 19). Substitution mutation analyses of mutants were performed and compared with the Wuhan-Hu-01 sequence using BioAider (version 1.314) (29). Phylogenetic analyses were conducted with IQ-Tree2, applying the maximum likelihood method with 1,000 bootstrap replicates using the GTR+F+R3 model by MFP ModelFinder (30). The trees were annotated and modified using Figtree (version 1.4.4) (https://github.com/rambaut/figtree/releases).

### Molecular dynamics simulation.

The complex structure of the SARS-CoV-2 S-protein RBD domain and human ACE2 was obtained from the Nation Microbiology Data Center (identifier [ID] NMDCS0000001) (PDB ID 6LZG) (31). Mutated amino acids of the SARS-CoV-2 RBD mutants were directly replaced in the model, and the bat/pangolin CoV RBD domain was modeled using SWISS-MODEL (32). Molecular dynamics simulation was performed using GROMACS 2019 with the following options and parameters: explicit solvent model, system temperature of 37°C, all-atoms optimized potentials for liquid simulations (OPLS/AA) force field, and LINCS restraints. With 2-fs steps, each simulation was performed at 10 ns, and each model was simulated three times to generate three independent trajectory replications. Binding free energy (Δ*G*) was calculated using the molecular mechanics Poisson-Boltzmann surface area (MM-PBSA) method (GitHub; https://github.com/Jerkwin/gmxtool), with the trajectories after structural equilibrium assessed using root mean square deviation (RMSD) (35, 36). To calculate the equilibrium dissociation constant (*K_D_*) and Δ*G*, the formula Δ*G* = RTlnK_D was used. Estimated Δ*G*s of the RBD mutants were normalized using the Δ*G* of the prototype strain, which was derived from experimental data.

### Expression of wild-type and mutant S proteins (V367F).

The SARS-CoV-2 prototype S gene lacking the furin cleavage site, TM, and cytoplasm domains was cloned into pNPM5 vector (Novoprotein, NJ, USA) and fused with a C-terminal His_6_ tag. The mutation was introduced by site-directed mutagenesis using the ClonExpress MultiS One Step cloning kit (Vazyme) and confirmed by PCR and sequencing. The wild-type and V367F constructs were transfected into HEK293 cells using polyethyleneimine. Since the S protein included a signal peptide in its N-terminal 14 amino acids, it was secreted into the medium. The expressed S proteins were purified from filtered cell supernatants with a Ni-nitrilotriacetic acid (NTA) column. Eluted protein solution was then dialyzed against phosphate-buffered saline (PBS; pH 7.4) for subsequent assays.

### Ligand-receptor binding ELISA.

Human ACE2 protein was immobilized onto a microtiter plate at 5 μg/ml (100 μl/well). Each S protein (prototype and V367F) was added as a ligand at different concentrations, ranging from 0.03 μg/ml to 10 μg/ml, and then incubated for 2 h at 37°C to allow receptor-ligand interaction. The ligand-receptor mixture was then washed three times. One hundred microliters of horseradish peroxidase (HRP) anti-His tag antibody (BioLegend, USA) (diluted 1:20,000) was added to each well and allowed to react for 1 h. After three washes, the signal was visualized using 3,3′,5,5′-tetramethylbenzidine (TMB) solution (Sigma-Aldrich, USA), and a microtiter plate reader recorded the optical density at 450 nm (OD_450_).

### Surface plasmon resonance experiments.

The SPR experiments were performed in a Biacore T200 instrument (GE, USA). For this, the SARS-CoV-2 S-proteins, either prototype or V367F, were immobilized on the sensor chip NTA (GE, USA), according to the manufacturer’s protocol. Human ACE2 protein was injected in each experiment at seven concentrations (6.25, 12.5, 25, 50, 100, 200, and 400 nM). For each cycle, the absorption phase lasted for 120 s and the dissociation phase lasted for 600 s. After each cycle, the chip was regenerated by washing with 350 mM EDTA and 50 mM NaOH for 120 s so that the chip was ready for the next round of S protein immobilization. Blank controls with 0 nM ACE2 were performed, with the blank signals subtracted from the cycle signals. All experiments were performed at 37°C. *K_D_* values were calculated by fitting the curves using the software provided with the instrument.

### Production and titration of SARS-CoV-2 pseudoviruses bearing V367F S protein.

The full-length S gene of SARS-CoV-2 strain Wuhan-Hu-1 (NC_045512.2) was cloned into a SARS-CoV-2 spike vector (PackGene, Guangzhou, China) and confirmed by DNA sequencing. Plasmid SARS-CoV-2 spike (G1099T), incorporating the V367F mutation in the S gene, was constructed by site-directed mutagenesis using the ClonExpress MultiS One Step cloning kit (Vazyme), as per the manufacturer’s protocol.

Generation of SARS-CoV-2 S HIV backbone pseudovirus was performed as previously described with some modifications (33, 34). Briefly, 293T cells, at approximately 70% to 90% confluence, were cotransfected with 9 μg of the transfer vector (pLv-CMV), 6 μg packaging plasmid (psPAX-lentiviral), and 6 μg envelope plasmid (pCB-spike or pCB-spikeV367F). Pseudoviruses were harvested at 48 h posttransfection and subsequently filtered into either Falcon or microcentrifuge tubes via a syringe-driven 0.45-μm filter. The harvested pseudoviruses were treated with recombinant DNase I (RNase-free) (TaKaRa, Japan) at 37°C for 10 min to eliminate carryover of plasmid DNA. Afterwards, the DNase I in the virus stock was inactivated at 95°C for 10 min after DNA digestion. Virus titration was measured by reverse transcription-quantitative PCR (RT-qPCR) targeting the WPRE gene of pseudoviruses using the Hifair III One Step RT-qPCR SYBR green kit (Yeasen), as per the manufacturer’s protocol. After reverse transcription (10 min at 42°C) and initial denaturation (5 min at 95°C), PCR amplification was performed for 40 cycles (15 s at 95°C, 60 s at 60°C). Primers were as follows: WPRE-F, 5′-CACCACCTGTCAGCTCCTTT-3′; WPRE-R, 5′-ACGGAATTGTCAGTGCCCAA-3′.

### SARS-CoV-2 spike-mediated pseudovirus entry assay.

To detect S variant-mediated viral entry, Vero E6 and Caco-2 cells (5 × 10^4^) grown in 24-well plates were infected with either S-V367 or S-F367-bearing purified pseudovirus (pretreated with DNase I) (1 × 10^6^ pseudovirus RNA copies). At 24 h and 48 h postinfection, the cells were washed three times, and the total DNA was extracted. Relative fold increase of infected virus titers was calculated according to the WPRE DNA copy number of the lentiviral proviruses measured by TB Green Premix Ex *Taq* II real-time PCR kit (TaKaRa). Data from all of the samples were obtained from three independent experiments, and each sample was tested in triplicates.
